# Intelligent Soft Sensor for Spindle Convective Heat Transfer Coefficient Under Varying Operating Conditions Using Improved Grey Wolf Optimization Algorithm

**DOI:** 10.3390/s25185806

**Published:** 2025-09-17

**Authors:** Jinxiang Pian, Gen Li

**Affiliations:** College of Electrical and Control Engineering, Shenyang Jianzhu University, Shenyang 110168, China; pianjx@sjzu.edu.cn

**Keywords:** spindle, convective heat transfer coefficients (CHTC), soft sensor, data-driven, improved grey wolf optimization (IGWO)

## Abstract

The thermal deformation of high-precision CNC machine tools has long been a significant barrier to improving machining accuracy. Accurately characterizing the thermal properties of the spindle, especially the convective heat transfer coefficients (CHTC), is essential for precise thermal analysis. However, due to the lack of dedicated instruments for directly measuring the CHTC, thermal analysis of the spindle faces substantial challenges. This study presents an innovative approach that combines multi-sensor data with intelligent optimization algorithms to address this issue. A distributed temperature monitoring network is constructed to capture real-time thermal field data across the spindle. At the same time, an improved Grey Wolf Optimization (IGWO) algorithm is employed to dynamically and accurately identify the CHTC. The proposed algorithm introduces an adaptive weight adjustment mechanism, which overcomes the limitations of traditional optimization methods in dynamic operating conditions. Experimental results show that the proposed method significantly outperforms conventional approaches in terms of temperature prediction accuracy across a broad operating range. This research provides a novel technical solution for machine tool thermal error compensation and establishes a scalable intelligent indirect measurement framework, even in the absence of specialized measurement instruments.

## 1. Introduction

In recent years, the prediction of the temperature field in the whole process of instantaneous and steady state of high-speed motorized spindle demands for enhanced machining efficiency and precision in manufacturing have driven the development of high-precision CNC machine tools towards higher speeds and greater intelligence. As a core functional component of CNC machine tools, the mechanical spindle plays a crucial role in determining overall machine performance and machining accuracy [1]. During high-speed rotation, however, significant heat is generated within the spindle due to friction and losses. This heat causes thermal deformation of the spindle itself and thereby introduces workpiece machining errors, representing a primary constraint on further improving machine tool precision [2]. The convective heat transfer coefficient (CHTC) is a key physical parameter governing the heat exchange process. It quantifies the efficiency of heat transfer between the spindle and its surrounding medium, and its accurate determination is essential for establishing high-fidelity thermal models [3]. Consequently, a deep understanding of the spindle’s heat transfer mechanisms and precise characterization of its thermal properties, particularly the CHTC, form the foundation for effective thermal error control and compensation in machine tools. Therefore, methods for determining the CHTC have been extensively studied across multiple fields.

However, the dynamic and complex nature of the CHTC, particularly their time-varying nonlinear characteristics under variable operating conditions, poses significant challenges for direct measurement. Although conventional heat flux sensors and non-contact infrared thermography can provide temperature distribution data, they cannot directly measure the CHTC, thus limiting the accuracy of thermal models. Conversely, empirical formula-based methods for calculating the CHTC are widely adopted due to their computational efficiency [4]. For example, one study proposed CHTC correlations based on environmental temperature [5], while another derived empirical formulas relating the CHTC to operating parameters using response surface analysis [6]. These empirical approaches, however, are highly dependent on assumed conditions, such as predefined flow boundaries and regimes. Consequently, under high-speed rotation or complex flow conditions, calculated results often exhibit significant deviations from actual values.

Advances in sensor and data fusion technologies have established multi-sensor temperature data acquisition as a crucial method for obtaining thermal information on spindles [7,8]. Deploying multiple temperature sensors enables high-precision reconstruction of the spindle thermal field distribution and real-time monitoring of its thermal state. Recent studies have attempted to estimate the CHTC indirectly by constructing heat transfer models through theoretical modeling and numerical simulation corrections [9,10]. For instance, in Ref. [8], the CHTC was treated as a nonlinear time-varying parameter and determined via data fitting, incorporating factors such as frictional torque, coolant, and environment temperature. Ref. [11] proposed a response surface optimization-based method to optimize the CHTC using experimental data. However, current research has not effectively established deep correlations between these multi-dimensional temperature datasets and critical thermal parameters like the CHTC, limiting their practical engineering application and generalizability.

On the other hand, the emergence of intelligent optimization algorithms offers a new approach for the dynamic identification of the CHTC. Specifically, Ref. [12] optimized the CHTC using a Genetic Algorithm (GA) to improve temperature prediction accuracy. Ref. [13] employed an Immune Algorithm (IA) to optimize the CHTC and combined this with a finite element thermo-mechanical coupling model to analyze the impact of thermal error on machining precision. In Ref. [14], the Lion Swarm Optimization (LSO) algorithm was used to optimize the spindle CHTC, where CHTC values were initially calculated using empirical formulas. Furthermore, Ref. [15] applied the Biogeography-Based Optimization (BBO) algorithm to optimize the CHTC of a motorized spindle and validated the thermal deformation prediction model using surface temperature data. Although these classical optimization algorithms have achieved some success in CHTC identification, they often suffer from limitations. Specifically, they tend to become trapped in local optima when dealing with multimodal problems or complex dynamic operating conditions, and their convergence speed is typically slow, making them unsuitable for real-time optimization requirements. In recent years, the Grey Wolf Optimizer (GWO) algorithm has gained widespread application in various optimization problems due to its strong global search capability. For instance, Ref. [16] utilized the GWO to optimize the CHTC, enhancing prediction accuracy and providing theoretical support for motorized spindle temperature control. Ref. [17] introduced a novel method for dynamically and accurately identifying actual CHTC values under varying operational conditions. Additionally, Ref. [18] proposed an intelligent CHTC identification optimization framework to further enhance the overall prediction accuracy. Furthermore, the study in Ref. [19] focused on selecting optimal temperature sensors and constructing a sophisticated POA-LSTM model for thermal error prediction.

In summary, current techniques for determining the CHTC face multiple challenges: on one hand, traditional online measurement instruments cannot meet the high-precision requirements under high-speed rotation and complex environments; on the other hand, empirical formula-based estimation methods exhibit limited accuracy under high-speed operating conditions. Although intelligent optimization algorithms show potential, the performance of existing algorithms in dynamic conditions still requires improvement. Moreover, effective fusion of multi-sensor data and accurate identification of thermal parameters remain key research foci. These technical bottlenecks not only hinder the precise acquisition of CHTC parameters but also limit the accuracy improvement and scenario adaptation of existing research in related fields such as heat dissipation structure design, thermal network modeling, and thermal error prediction. Based on the core requirements for accurate CHTC identification—meeting the thermal boundary conditions for heat dissipation structure design, addressing model errors caused by fixed CHTC assumptions, and bridging the gap in physical accuracy of underlying thermal models in the pre-modeling stage—this study proposes an intelligent soft-sensing method based on an IGWO to systematically address these challenges. The method incorporates an adaptive weight adjustment mechanism and a dynamic disturbance strategy. The former adjusts search weights in real time according to operational conditions, while the latter effectively prevents the algorithm from falling into local optima. Together, they significantly enhance the global search capability and convergence speed of the optimization algorithm under complex dynamic conditions, aligning with the aforementioned technical need for “dynamic identification of actual CHTC”. Furthermore, this study constructs a multi-channel temperature sensing network to comprehensively collect thermal response data, which is deeply integrated with the IGWO algorithm. This integration ultimately enables dynamic and accurate identification of spindle CHTC, technically achieving the research objective of “ensuring the physical accuracy of the thermal model”. Experimental results demonstrate that the proposed method significantly outperforms traditional methods in temperature prediction accuracy under varying conditions. This not only validates the effectiveness of dynamic CHTC identification but also provides a transferable technical framework for thermal error compensation in machine tools, supporting the core value of “enhancing the robustness and generalization capability of subsequent thermal error models”.

The remainder of this article is organized as follows. Section 2 analyzes the spindle CHTC problem description. Section 3 introduces a soft sensor method for the CHTC of mechanical spindles based on an IGWO. Section 4 discusses experimental results. Finally, Section 5 concludes this article.

## 2. Spindle CHTC Problem Description

### 2.1. Mechanical Spindle Structure

**Figure 1:**
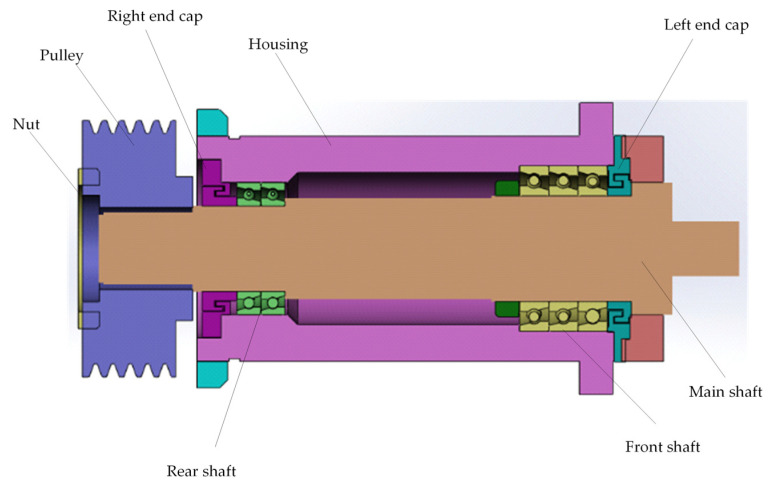
The structure of a typical CNC lathe’s mechanical spindle.

Figure 1 shows the structure of a typical CNC lathe spindle, which is the core transmission component of the machine. Its performance directly determines the machining accuracy, speed range, and operational stability of the lathe. The spindle system is composed of the shaft, housing, front and rear angular contact ball bearings, and bearing end caps, all tightly assembled. The selected angular contact ball bearings are capable of bearing both radial and axial loads. By precisely adjusting the preload, they effectively suppress axial movement during high-speed operation, ensuring the spindle maintains high precision and stability, which is crucial for the overall performance of the system. During high-speed rotation, friction, thermal flow, and load generate significant heat, which is exchanged with the spindle, leading to thermal deformation.

In spindle thermal characteristic analysis, the CHTC is a crucial physical parameter that determines the heat exchange efficiency between the spindle and its surrounding medium. Therefore, accurately obtaining the CHTC is key to solving thermal deformation problems. However, in practical applications, directly measuring the convective CHTC of the spindle faces several challenges, which are mainly reflected in the following aspects:

- **Complexity of CHTC’s physical characteristics**: The value of CHTC is influenced not only by inherent factors such as the surface roughness and thermal conductivity of the spindle material but also by factors such as the fluid flow state, temperature distribution, and heat transfer conditions at the contact surface. These variables are interrelated, making the calculation and measurement of CHTC extremely complex. As a result, it is difficult to obtain accurate values through traditional direct measurement methods.
- **Limitations of measurement methods:** Traditional methods for measuring CHTC typically rely on devices like heat flux sensors or infrared thermometers. However, these methods mainly depend on indirect measurements of surface temperature or heat flow and cannot directly capture the heat exchange process between the spindle and its surrounding medium. Moreover, the complex surface shape of the spindle, combined with the constantly changing fluid flow conditions during high-speed operation, limits the accuracy and applicability of traditional methods. They often fail to reflect the actual heat exchange efficiency under working conditions.
- **Dynamics and variability of operating conditions:** The operating conditions of the spindle vary significantly across different machining tasks, including factors such as speed, load, and environmental temperature. These fluctuations lead to dynamic changes in the fluid state, heat transfer mode, and the CHTC. As a result, traditional static measurement methods struggle to track and capture these changes in real-time, causing delays and inaccuracies in CHTC measurements.
- **Challenges in high-temperature and high-flow environments**: During high-speed operation, heat exchange between the spindle surface and the fluid occurs in a high-temperature, high-flow environment. This makes it difficult for traditional measurement methods, such as direct placement of thermocouples or heat flux sensors, to accurately capture the CHTC. Furthermore, these methods may damage the equipment or interfere with the spindle’s operation. Therefore, accurately measuring the CHTC under these extreme conditions remains a significant technical challenge.

### 2.2. Analysis of Factors Affecting the CHTC

(1) The CHTC of the spindle exhibits an unknown, complex nonlinear relationship with both its rotational speed and the environmental temperature.

During convective heat transfer from a mechanical spindle, the CHTC depends on both the rotational speed and the environmental temperature. Furthermore, the relationship between these factors is characterized by complex nonlinearity. Consequently, this multi-factor interaction makes predicting CHTC variations significantly more challenging.

Specifically, the primary heat source within the spindle originates from bearing frictional heat. As the rotational speed increases, this frictional heat also increases. This results in a larger temperature difference (*ΔT*) between the spindle surface and the surrounding air, thereby enhancing the heat transfer efficiency. Therefore, the spindle’s CHTC shows a positive correlation with rotational speed. However, this relationship is not a simple linear increase. Instead, as speed rises, the flow regime transitions from laminar to turbulent. Correspondingly, empirical formulas for the CHTC under these two distinct flow states are provided in Ref. [20].

CHTC in Laminar Flow:

(1) H=Nu·λl=0.664 Re1/2 Pr1/3λ/l

CHTC in Turbulent Flow:

(2) H=Nu·λl=0.037 Re1/2 Pr1/3λ/l

(3) Re=v×d/μ

(4) μ=2πrn/60

where *H* is the CHTC (W/(m^2^·K)); *N_u_* is the Nusselt number; Re is the Reynolds number; *λ* is the thermal conductivity of air; *l* is the characteristic length; *n* is the rotational speed; *r* is the bearing radius; *d* is the bearing diameter; *μ* is the kinematic viscosity of air; *v* is the tangential velocity; and Pr is the Prandtl number. It can be observed that the enhancement of the CHTC becomes more significant during the transition from laminar to turbulent flow, exhibiting a nonlinear increasing trend. Moreover, increased rotational speed further nonlinearly enhances the heat exchange process via centrifugal force effects and surface geometric perturbations, among other factors.

On the other hand, the spindle CHTC is not only influenced by rotational speed but also closely related to the environment temperature (*T_i_*). This is because the environmental temperature determines the temperature difference between the spindle surface and the surrounding air, which is the driving force for heat transfer. When the environmental temperature is low, this temperature difference is large, resulting in more efficient heat exchange between the spindle and air, and consequently a higher CHTC. Conversely, when the environmental temperature increases, the temperature difference decreases, leading to reduced heat transfer efficiency and thus a lower CHTC. Moreover, changes in environmental temperature not only alter the temperature difference. They also indirectly affect fluid flow characteristics by influencing the physical properties of air (such as density, viscosity, and specific heat capacity). This further complicates the nonlinear relationship between CHTC and environmental temperature.

(2) The CHTC varies at different positions along the spindle.

During operation, different regions of the spindle experience distinct thermal environments and heat transfer mechanisms, resulting in non-uniform CHTC values. Firstly, heat exchange between the spindle surface and the surrounding air critically influences the spindle’s overall cooling efficiency. Significant variations exist between different spindle sections regarding temperature distribution, airflow effects, and heat source intensity. Consequently, analyzing the CHTC at these locations reveals their specific heat exchange characteristics. Secondly, friction heat from internal components, such as bearings, constitutes a major heat source. This heat dissipates primarily through convection via air gaps and the surrounding air; analyzing heat transfer at these points is essential. Furthermore, the conductive and convective properties of the spindle housing and bearings directly impact the spindle’s overall thermal stability. Therefore, to comprehensively understand the spindle’s thermal management characteristics, the convective CHTC at the following seven key positions was specifically selected for investigation in this study.

1. **Convection heat transfer coefficient H1 at the rear shaft pulley and environment air**: This region is an exposed external part of the spindle shaft, directly contacting environment air. It is significantly influenced by airflow and environmental temperature; thus, analyzing its heat transfer characteristics is essential.
2. **Convection heat transfer coefficient H2 at the right end face of the rear bearing and the air gap**: This interface connects the spindle interior to the external environment. Frictional heat from the bearing is dissipated here, exchanging heat with air via the air gap. Understanding heat transfer at this location is critical for evaluating bearing heat dissipation performance.
3. **Convection heat transfer coefficient H3 at the intermediate bearing and the air gap:** The intermediate bearing is a critical spindle component. Subject to frictional heat and rotational speed, heat transfer between the bearing and the surrounding air gap is vital for overall spindle thermal management.
4. **Convection heat transfer coefficient H4 at the left end face of the rear bearing and the air gap:** This end face also participates in heat exchange, conducting heat to the air through the air gap. It influences the spindle’s thermal distribution and stability. Analysis here further clarifies the heat dissipation capacity from the bearing heat source.
5. **Convection heat transfer coefficient H5 at the front shaft shoulder and environment air:** Contacting environment air, the front shaft shoulder represents another key heat dissipation zone. Particularly during high-speed operation, its heat transfer characteristics play a pivotal role in spindle temperature control.
6. **Convection heat transfer coefficient H6 at the housing and the air gap:** The spindle housing is isolated from direct environmental air contact. The presence of the air gap necessitates a focused analysis of heat transfer characteristics at this interface. Heat exchange efficiency between the housing and air gap is crucial for the spindle’s overall thermal performance.
7. **Convection heat transfer coefficient H7 at the housing and environment air:** Direct contact between the housing and environment air affects the spindle’s heat exchange efficiency, especially under elevated temperatures. Heat transfer characteristics here are essential for maintaining operational stability and extending service life.

Collectively, the CHTC for these seven critical locations is represented by Equation (5):

(5) $$\left(H1,H2,H3,H4,H5,H6,H7)=F(n,T_{i}\right)$$

**Figure 2:**
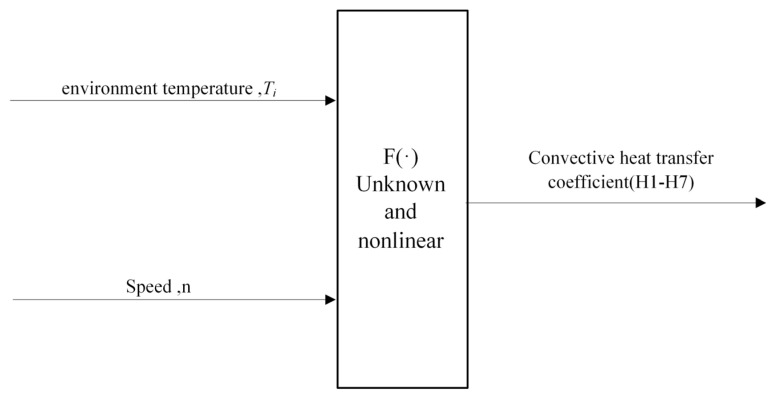
Input–output relationship diagram of the spindle CHTC model.

In the equation, *F*(·) represents an unknown nonlinear relationship, *H*1–*H*7 denote the CHTC, *n* is the spindle speed, and *T_i_* represents the environment temperature. The input–output relationship is illustrated in Figure 2. When the environment temperature *T_i_* and spindle speed *n* vary, the CHTCs must be determined appropriately. This ensures that the temperature distribution calculated by the model based on these CHTCs closely matches actual measurements. Essentially, this constitutes a scientific problem of nonlinear optimization under dynamic conditions.

In summary, when the environmental temperature *T_i_* and s speed *n* vary, the rational determination of the CHTC to align the simulated temperature distribution with reality is, fundamentally, a nonlinear dynamic optimization problem.

## 3. Intelligent Soft Sensor for Spindle CHTC Under Varying Operating Conditions Using IGWO

In order to achieve an intelligent soft sensor for spindle CHTC, a multi-sensor temperature network was first established to comprehensively collect temperature data from different key locations on the spindle. Due to significant spatial variations in temperature distribution during high-speed operation, a single sensor cannot adequately capture the thermal state changes across the entire spindle. Therefore, a temperature sensor network incorporating 32 detection channels was deployed. These sensors were strategically positioned near the front and rear bearings, as well as in other regions of the spindle housing. This configuration ensures comprehensive monitoring of temperature fluctuations, thereby providing richer foundational data for CHTC calculation.

Moreover, spindle speed and environmental temperature are key factors influencing the CHTC. Spindle speed directly affects the relative motion between the spindle surface and the fluid, and it determines the flow regime during heat exchange, thereby impacting heat transfer efficiency. Meanwhile, environmental temperature governs the spindle’s heat exchange capacity with the surrounding medium. Crucially, temperature variations under different operating conditions significantly affect the CHTC. Consequently, acquiring this data is vital for accurately evaluating the CHTC. These parameters provide essential inputs for soft-sensing models and offer deeper insights into the spindle’s heat exchange characteristics under varying operational states.

Meanwhile, the soft-sensing of the CHTC is inherently a nonlinear optimization problem. To address this, we introduce an IGWO. As a robust global optimization algorithm, GWO effectively identifies optimal solutions within complex nonlinear relationships among multiple variables and features, and accelerates convergence. As reported in [21], an optimization algorithm was employed to address nonlinear problems in short-term wind power forecasting, and in [22], the GWO was applied to ore particle size detection. Similarly, [23] utilized the GWO to address nonlinear optimization problems in asset management and predictive maintenance. Its effectiveness in practical engineering is proven by its application to similar nonlinear optimization challenges like mobile robot path planning, as demonstrated in [24,25]. Leveraging this advantage, the improved GWO further enhances prediction accuracy for CHTC soft-sensing. Crucially, it enables real-time adjustments to the search strategy during dynamic operating conditions, ensuring accurate CHTC estimation.

### 3.1. Soft Sensor Modeling Strategy

**Figure 3:**
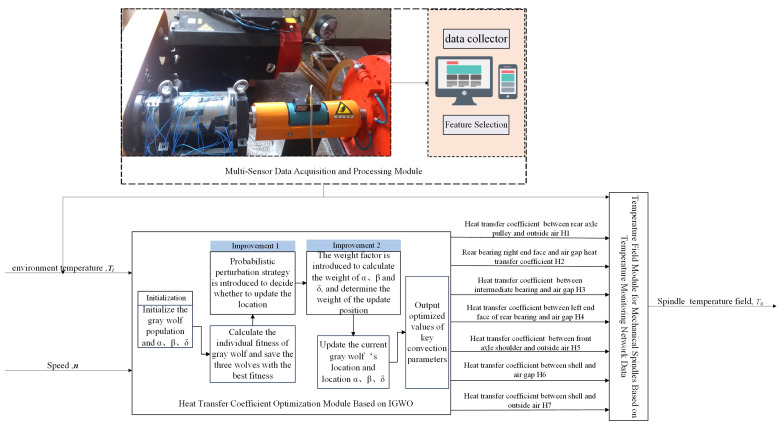
Soft sensor of CHTC for mechanical spindle.

Based on the preceding analysis, this study proposes a soft sensor strategy for CHTC estimation, as illustrated in Figure 3. This approach integrates multi-sensor network data with an IGWO. The proposed strategy comprises three key components: a multi-sensor data acquisition and processing module; an optimization module for CHTC based on an IGWO; and a temperature field module for the mechanical spindle, utilizing data from the temperature monitoring network. The multi-sensor data acquisition and processing module collects data from the temperature sensor network, spindle speed *n*, and environment temperature *T_i_*, conducting feature selection. Then, the convection heat-transfer coefficient optimization module, based on an IGWO, takes the environment temperature *T_i_* and spindle speed *n* as inputs to dynamically optimize the seven-spindle convection CHTC (*H*1–*H*7). Finally, the mechanical spindle temperature field module, using data from the temperature monitoring network, takes the seven optimized CHTC (*H*1–*H*7) and the environment temperature *T_i_* as inputs to calculate and output the mechanical spindle temperature field *T*_0_.

### 3.2. Soft Sensor Algorithm

#### 3.2.1. Improved Grey Wolf Optimizer

**Figure 4:**
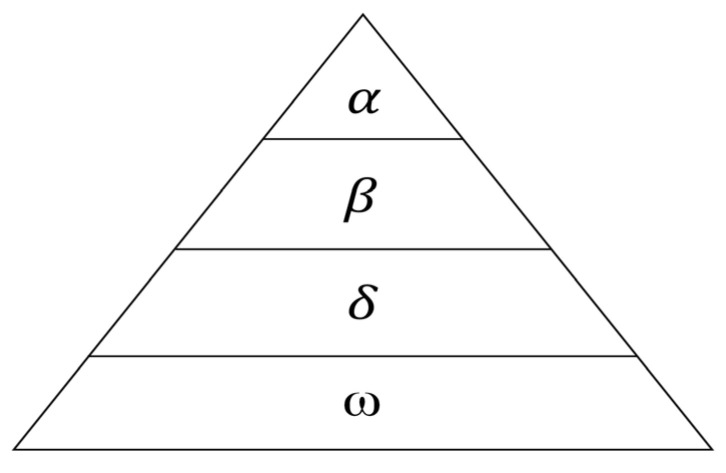
Schematic diagram of grey wolf hierarchy.

The GWO algorithm simulates the social hierarchy and hunting behavior of grey wolf packs. Grey wolves are social animals that typically form packs of around a dozen individuals, maintaining a strict dominance hierarchy. As illustrated in Figure 4, the pack is structured into four levels: *α*, *β*, *δ*, and *ω* wolves. The *α* wolves are the leaders, responsible for decisions regarding activities like hunting, resting locations, and food distribution. *β* wolves assist the *α* and often serve as potential successors. *δ* wolves support the *α* and *β* in carrying out tasks. *ω* wolves comprise the remaining individuals, following the orders of the higher-ranked wolves and primarily maintaining pack harmony. During hunting, the *α*, *β*, and *δ* wolves approach the prey directly. Meanwhile, the ω wolves follow and assist in encircling the target. Once the prey is surrounded, the pack attacks. In the GWO, the individual with the best fitness is designated the *α*. The two individuals with the next best fitness values become the *β* and *δ*, respectively, while the remaining individuals are considered the *ω*.

Corresponding to the function optimization problem in the GWO algorithm, the individual with the highest fitness is designated *α*. The two next best individuals are defined as *β* and *δ*, respectively, and the remaining individuals are *ω*. During the hunting process, the grey wolf pack is led by *α*, assisted by *β* and *δ*, with the *ω* wolves following. This process primarily involves three steps: encircling, hunting, and attacking. These steps ultimately lead to capturing the prey (the global optimum solution) with a high success rate.

Specifically, the grey wolves first encircle the prey. This encircling behavior is modeled mathematically as:

(6) $$D_{0}=\left|C\cdot X_{p}(t)-X(t)\right|$$

(7) $$X(t+1)=X_{p}(t)-A\cdot D_{0}$$

(8) $$A^{2}=2a\cdot r_{1}-a$$

(9) $$C=2r_{2}$$

Herein, *D*_0_ denotes the distance metric between the grey wolf individual and the prey position, adjusted by coefficient ***C***; *X_p_*(*t*) represents the position of the prey; and *X*(t) signifies the position of the grey wolf individual at iteration t. ***A*** and ***C*** are coefficient vectors, *r_1_* and *r_2_* are random vectors within [0,1], and *a* is the control parameter.

Subsequently, the wolf pack enters the encircling phase during which the positions of the grey wolf individuals are updated under the guidance of the *α*, *β*, and *δ* wolves. This process is mathematically expressed as follows [26]:

(10) $$D_{\alpha }=\left|C_{1}\cdot X_{\alpha }-X(t)\right|$$

(11) $$D_{\beta }=\left|C_{2}\cdot X_{\beta }-X(t)\right|$$

(12) $$D_{\delta }=\left|C_{3}\cdot X_{\delta }-X(t)\right|$$

(13) $$X_{1}=X_{\alpha }-A_{1}\cdot D_{\alpha }$$

(14) $$X_{2}=X_{\beta }-A_{2}\cdot D_{\beta }$$

(15) $$X_{3}=X_{\delta }-A_{3}\cdot D_{\delta }$$

(16) $$X(t+1)=(X_{1}+X_{2}+X_{3})/3$$

Finally, the grey wolf pack attacks to achieve the objective of capturing the prey. This attack behavior is primarily governed by the parameter **a** in Equation (8), which linearly decreases from 2 to 0 throughout iterations. When |A| ≤ 1, the wolves converge to attack the prey, corresponding to a local search. Conversely, when |A| > 1, the wolves disperse to perform a global search.

However, while the standard GWO drives the population toward promising regions by averaging the positions of the *α*, *β*, and *δ* wolves, this simplistic update rule overlooks individual contributions during the collective hunting process. As a result, the guidance provided by the leaders may lack adaptive nuance, often causing premature convergence and reducing population diversity—especially in later iterations when all individuals cluster closely around suboptimal solutions. Once trapped in local optima, the algorithm exhibits limited capacity for escape.

To mitigate these inherent limitations, it is critical to not only introduce perturbations but to do so in a manner that aligns with the search behavior and balances exploration with exploitation. Therefore, this paper proposes two targeted modifications: an Introduction of a Weight Factor that differentiates the influence of each leader wolf based on their fitness, and a probabilistic perturbation strategy that injects adaptive noise into the update process. These enhancements are specifically designed to preserve diversity, facilitate escape from local optima, and improve convergence accuracy—making them particularly suitable for solving complex optimization problems where the standard GWO often underperforms.

(1) Introduction of a Weight Factor

To address the contribution of solutions to the optimal solution in the GWO algorithm, this study proposes a novel framework for adjusting a weight factor. Given the time-varying nature of the coefficient vectors ***A*** and ***C***, which govern the search behavior, the designed weight factor employs a dynamic nonlinear adjustment mechanism. This mechanism ensures synergistic adaptation with the evolutionary process of the algorithm’s parameters. Therefore, the proposed weight factor is intrinsically linked to the coefficient vectors **A** and **C**. Specifically, in the basic GWO algorithm, the values of ***A_1_***, ***A_2_***, ***A_3_,*** and ***C_1_***, ***C_2_***, ***C_3_*** differ. To ensure correlated updates of the weight factor, ***A_1_***, ***A_2_***, and ***A_3_*** are designed to be identical, and likewise, ***C_1_***, ***C_2_***, and ***C_3_*** are designed to be identical.

(17) $$A_{1}=A_{2}=A_{3}=2a\cdot r_{1}-a$$

(18) $$C_{1}=C_{2}=C_{3}=2r_{2}$$

Therefore, the designed position vector weighting factors are:

(19) $$\omega_{1}=\frac{\left|A_{1}\cdot C_{1}\right|}{\left|A_{1}\cdot C_{1}\right|+\left|A_{2}\cdot C_{2}\right|+\left|A_{3}\cdot C_{3}\right|}$$

(20) $$\omega_{2}=\frac{\left|A_{2}\cdot C_{2}\right|}{\omega_{1}+\left|A_{2}\cdot C_{2}\right|+\left|A_{3}\cdot C_{3}\right|}=\frac{3\left|A_{1}\cdot C_{1}\right|}{1+6\left|A_{1}\cdot C_{1}\right|}$$

(21) $$\omega_{3}=\frac{\left|A_{3}\cdot C_{3}\right|}{\omega_{1}+\omega_{2}+\left|A_{3}\cdot C_{3}\right|}=\frac{18{\left|A_{1}\cdot C_{1}\right|}^{2}+3\left|A_{1}\cdot C_{1}\right|}{1+18\left|A_{1}\cdot C_{1}\right|+18{\left|A_{1}\cdot C_{1}\right|}^{2}}$$

Therefore, we derive the new position update formula as:

(22) $$X(t+1)=\frac{\omega_{1}X_{1}+\omega_{2}X_{2}+\omega_{3}X_{3}}{3}$$

(2) Probabilistic Perturbation Strategy

This paper introduces a novel probabilistic perturbation strategy. Its purpose is to enhance population diversity during the later stages of algorithm optimization. This prevents the algorithm from converging prematurely to local optima. Notably, similar probabilistic perturbation techniques have already been employed in existing optimization algorithms. Thus, we designed a simple yet effective perturbation probability formula:

(23) $$P=\frac{(G-1)e^{(t-1)/k}}{4G}$$

In the algorithm, the perturbation probability *P*, dimension *G*, and maximum iteration count *k* form a synergistic parameter framework. During the early optimization stage, a lower *P* value facilitates rapid convergence towards the global optimum. Conversely, in the later stages, increasing *P* effectively maintains population diversity to escape local optima. Typically, *P* is set to a relatively large, fixed value. As the iteration count approaches the maximum t→*T*, the perturbation probability $P=\frac{(G-1)e^{(t-1)/k}}{4G}\to \frac{(1-1/G)e}{4}$ the perturbation formula for an individual grey wolf agent is given by:

(24) $$M(t+1)=lb+r_{3}\cdot (ub-lb);r_{3}<p$$

Here, *M* denotes the individual after perturbation. The lower and upper bounds of the individual position in the GWO are represented by *lb* and *ub*, respectively. Additionally, *r_3_* is a randomly generated vector with elements uniformly distributed between 0 and 1. Subsequently, the perturbed grey wolf individual is updated via a greedy mechanism. The update formula is given as follows:

(25) $$X(t+1)=\left\{\begin{array}{l} M(t+1),f(M(t+1))<f(X(t+1))\\ X(t+1),\mathrm{others} \end{array}\right.$$

where *f*(*M*(*t* + 1)) and *f*(*X*(*t* + 1)) denote the objective function values of the perturbed individual *M* and the grey wolf individual *X* at the (t + 1)th generation, respectively.

#### 3.2.2. Validation of Effectiveness for the IGWO

To validate the optimization performance of the IGWO, four representative benchmark functions were selected for comparative experiments: unimodal functions F1 and F2, and multimodal functions F7 and F9. Details for each function (expression, dimensionality, search range) are provided in Table 1. This selection serves specific purposes: unimodal functions primarily assess an algorithm’s local search capability and convergence speed, effectively reflecting its efficiency in approximating the optimal solution within smooth search spaces. Conversely, multimodal functions, with their complex landscapes, rigorously test global search ability by evaluating the algorithm’s capacity to escape local optima and locate the global optimum.

In terms of computational cost, although the introduction of a weight factor and probabilistic perturbation strategy in IGWO increases the complexity per iteration compared to the standard GWO, this overhead is justified by a significant reduction in the number of iterations required to reach high-quality solutions. The improved convergence behavior mitigates the additional per-iteration cost, leading to competitive overall computational efficiency.

**Figure 5:**
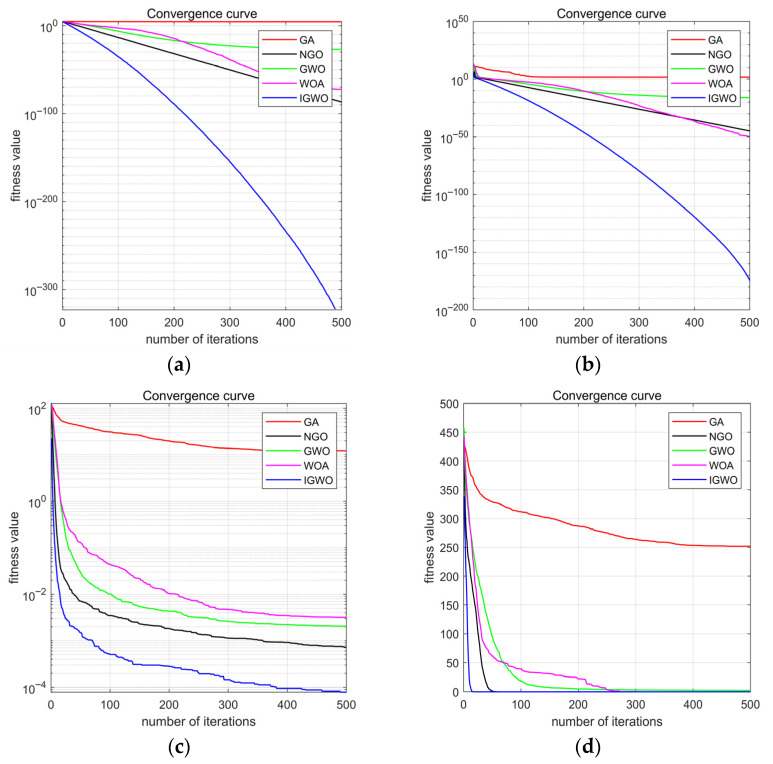
Comparison of test results based on benchmark functions: (**a**) optimization curve based on test function F1, (**b**) optimization curve based on test function F2, (**c**) optimization curve based on test function F7, (**d**) optimization curve based on test function F9.

The experimental results presented in Figure 5 demonstrate that during the optimization of unimodal functions F1 and F2, the IGWO algorithm exhibited superior performance from the initial iterations. Its fitness value decreased significantly faster than those of GWO, GA, WOA, and NGO. Consequently, IGWO approached or reached the theoretical optimum in fewer iterations, indicating enhanced local exploitation ability and convergence efficiency. For the multimodal functions F7 and F9, IGWO’s advantage was even more pronounced. It consistently escaped local optima and located the global optimum more accurately. The final fitness values achieved by IGWO were notably lower than those of the other algorithms, confirming its superior global exploration capability in complex search spaces.

Notably, the computational expense associated with IGWO remains manageable, as the increase in per-iteration cost is linear with respect to population size and problem dimension—similar to other population-based algorithms. The efficient implementation of the weight and perturbation mechanisms ensures that the algorithm remains scalable and practical for medium- to high-dimensional problems.

In summary, for both unimodal and multimodal functions, the IGWO algorithm outperformed the comparative algorithms in terms of convergence speed and search accuracy. These results demonstrate that the introduced weight factor and probabilistic perturbation strategy effectively enhance the algorithm’s optimization performance, enabling more robust and efficient search capabilities across diverse optimization problems, with only a modest and acceptable increase in computational cost.

#### 3.2.3. Optimization of CHTC Based on Improved Grey Wolf Algorithm

In the GWO, the prey position represents a solution within the optimization space, while the pack of wolves denotes all possible solutions. The wolves search for the optimal solution through the processes of searching, surrounding, and attacking the prey. The correspondence between the modified GWO and the optimization problem is summarized in Table 2.

1. Search Procedure

(1) Initialization: At the start of the search, each gray wolf represents a candidate CHTC solution. The initial positions of the wolf population, denoted as *X*(0) (*i* = 1, 2, …, *N*), are determined. The time step is set to *t* = 0.
(2) Fitness Evaluation and Ranking: The fitness of all gray wolves (CHTC values) is evaluated. Subsequently, these values are sorted in ascending order. The three wolves with the highest fitness (representing the best CHTC values) are identified as *α*, *β*, and *δ*. Their position coordinates are recorded as *X_α_*(*t*), *X_β_* (t), and *X_δ_*(*t*), respectively.
(3) Position Update: For each CHTC value (gray wolf), the coefficient vectors A and C are first calculated using Equations (17) and (18). Then, Equations (19)–(22) are applied to determine the new position *X*(*t* + 1) of the wolf at the next time step t + 1.
(4) Termination and Update Check: The algorithm terminates if a stopping criterion is met (reaching the maximum iteration count or desired accuracy). In this case, *X_α_* (t) is output as the global optimal solution. Otherwise, *t* is incremented (*t* = *t* + 1), and the process returns to Step (2). Additionally, if a specific CHTC value fails to update after *k* consecutive iterations, it is discarded as having converged to a local optimum.

2. Fitness Function

This study investigates the spindle’s transient temperature field, denoted as *T*_0_. As this field function depends on both the spatial domain *Ω* and the time domain t, *T*_0_ is difficult to solve directly. Hence, the finite element method (FEM) is employed to solve the governing equation for *T*_0_.

Since the time and spatial domains are decoupled, the differential equation governing the transient heat transfer problem for the 3D spatial field function and its boundary conditions is first transformed into an equivalent integral form. Subsequently, the spatial domain *Ω* is discretized into *x* finite elements. This process yields the FEM solution equation set, given by Equation (26).

(26) $$E\dot{T}+KT^{\prime }=Z$$

The above equation is a system of linear ordinary differential equations with time *t* as the independent variable, where *E* denotes the heat capacity matrix, *K* denotes the thermal conductivity matrix, and *Z* denotes the temperature load matrix, $T^{\prime }$ the node temperature array, $\dot{T}(=dT/dt)$ the derivative array of node temperature concerning time, matrices *K*, *E*, and *Z* are as follows [27]:

(27) $$K_{ij}=\int_{\Omega^{e}}k(\frac{\partial N_{i}}{\partial x}\frac{\partial N_{j}}{\partial x}+\frac{\partial N_{i}}{\partial \nu }\frac{\partial N_{j}}{\partial \nu }+\frac{\partial N_{i}}{\partial z}\frac{\partial N_{j}}{\partial z})d\Omega +\int_{S_{i}^{e}}H_{i}N_{i}N_{j}dS$$

(28) $$E_{ij}=\int_{\Omega^{e}}\rho cN_{i}N_{j}d\Omega$$

(29) $$Z_{i}=\int_{\Omega^{e}}\rho QN_{i}d\Omega +\int_{S_{i}^{e}}h_{i}T_{b}N_{i}dS$$

The transient temperature response was solved using the direct integration method with Equation (29), yielding:

(30) $$(E/\Delta t+\theta K)T_{n+1}=[E/\Delta t-(1-\theta )K]T_{n+1}+(1-\theta )Z_{n}+\theta Z_{n+1}$$

In the equation, *∆t* represents the time step. θ∈(0-1) Using the above equation, the instantaneous values *T_x_(t)* at each spindle node can be recursively determined starting from *t* = 0. Thus, *T_y_* = [*T*_1_(∆t), *T*_2_(2∆t), *T*_2_(∆t)].

In the IGWO, it is generally considered that a larger population size should correspond to a higher fitness value, implying that the algorithm should strive for increased fitness. For both minimization and maximization problems commonly encountered in optimization, fitness functions must be constructed accordingly. Let the objective function be denoted as *f_i_*. For minimization problems, the fitness function *fit_i_* is typically defined as a transformation of the objective function, as shown in Equation (31). In contrast, for maximization problems, the fitness function is often taken directly as the objective function value itself. Since this study addresses a minimization problem, the fitness function defined in Equation (32) is adopted for computation.

(31) $$fit_{i}=\frac{1}{1+f_{i}}$$

(32) $$f_{i}=\frac{\Sigma_{i=1}^{100}\sqrt{{\left(T_{S}-T_{y}\right)}^{2}}}{100}$$

where *T*_s_ is the measured temperature and *T*_y_ is the temperature obtained from the finite element (FE) simulation.

### 3.3. Implementation Procedures for CHTC Soft Sensor

**Figure 6:**
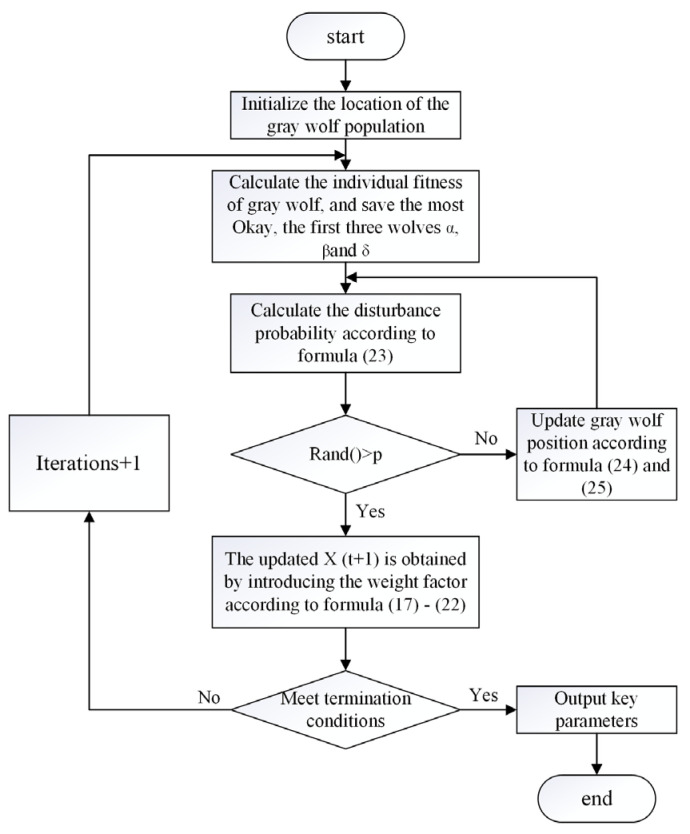
Flowchart of the CHTC soft sensor based on the IGWO.

As shown in Figure 6, the aforementioned IGWO algorithm was employed for soft sensing of the machine tool spindle CHTC, which involves the following steps.

Step 1: Population Initialization. Set the total number of CHTCs *D,* the maximum iteration count *k*, and the control parameter *a*. Define the search boundaries for the problem and randomly generate the initial CHTC solutions *x_i_* (where i = 1, 2, …, SN) within this range.

Step 2: Fitness Evaluation. Input these solutions into the finite element analysis (FEA) model for temperature field simulation. Calculate the temperature values at specific experimental positions. Compare these simulated temperatures with the experimentally measured values. Use this comparison to evaluate the fitness (*fit_i_*) of each initial solution and check if the termination criteria are met.

Step 3: Solution Check. If the fitness of all CHTC solutions is greater than 0.5, output the current CHTC values as the final optimized result. Otherwise, if *fit_i_* < 0.5, proceed to Step 4.

Step 4: Perturbation Probability. Calculate the perturbation probability *p* using Equation (23). If a random number rand() > *p*, continue execution. Otherwise, return to Step 4.

Step 5: Position Update. Introduce a weight factor and update the CHTC positions using Equations (19)–(22) to obtain new solutions.

Step 6: Best Solution Update. Record the current best solution.

Step 7: Termination Check. Check if the termination criteria are met. If met, terminate the iteration and output the current best solution. Otherwise, return to Step 2 to continue the search.

## 4. Experimental Studies

### 4.1. Experimental Platform and Data Description

**Figure 7:**
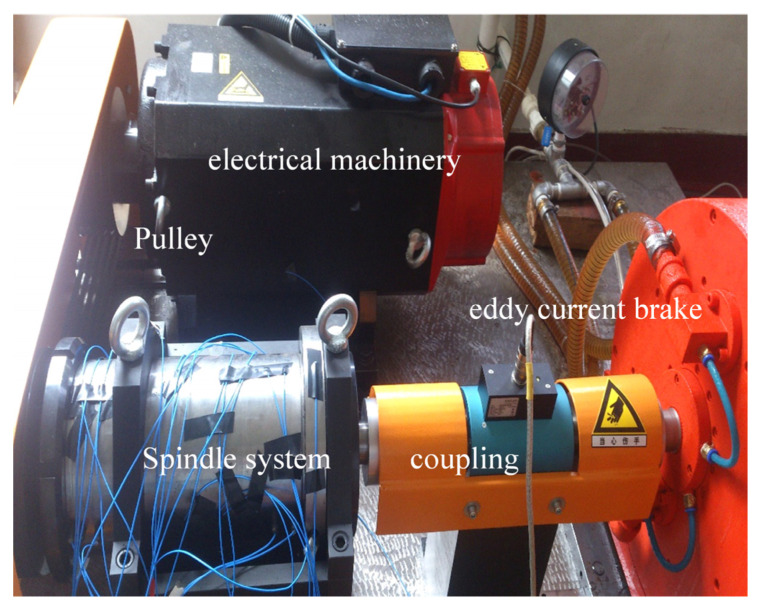
Automatic testing system for mechanical spindle performance characteristics.

Experimental research was conducted using an automatic mechanical spindle characteristic test apparatus, as shown in Figure 7. The load on the spindle was adjusted by varying the excitation current of the eddy current loader. This allowed measurement of the spindle’s torque, rotational speed, and power output under both no-load and loaded conditions. Analysis of the experimental data indicates that the spindle temperature rises significantly during sustained high-speed rotation. This temperature increase may adversely affect machining accuracy. Therefore, using this mechanical spindle as an example, we conducted an experimental study to validate the proposed CHTC soft-sensing model.

(1) Experimental Design

The test spindle was interconnected via couplings to a torque/speed sensor and a brake. A drive motor rotated the spindle through a belt transmission system to simulate actual operating conditions. The load was controlled by adjusting the brake excitation current. The spindle system, dynamometer control, and data acquisition systems operated independently to facilitate operation and data collection. To ensure measurement accuracy and repeatability, the torque/speed sensor was calibrated before the tests, and measurement uncertainties were quantified. External disturbances (e.g., environmental temperature fluctuations and mechanical vibrations) were minimized by conducting the experiments in a controlled laboratory environment. The spindle was returned to environmental temperature (20 °C ± 0.5 °C) before each test, with a 5 h interval between tests to ensure thermal equilibrium. Temperature data were acquired at spindle speeds of 3000, 4000, 5000, 6000, and 7000 rad/min. Each test lasted 60 min, as spindle temperatures stabilized after approximately 25–30 min. All tests were repeated three times to verify repeatability.

(2) Multi-Sensor Temperature Monitoring Network

**Figure 8:**
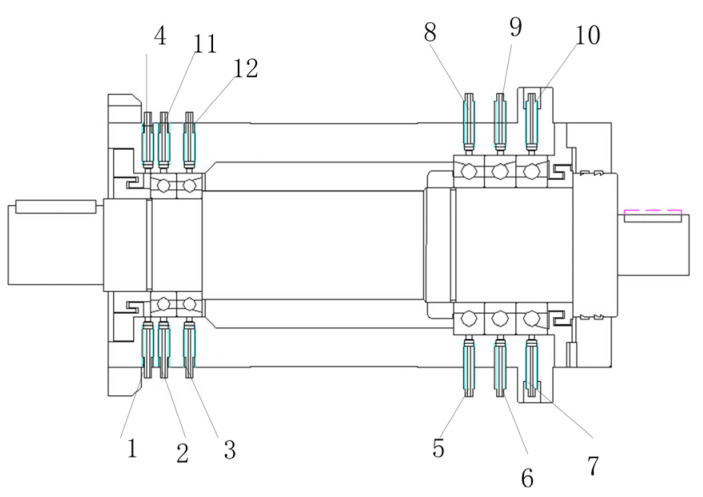
Measurement points 1–12 layout diagram.

The test system featured 32 data acquisition channels. Considering that friction between the spindle and bearings is the primary heat source, 12 calibrated temperature sensors were installed at the front and rear bearing locations, positioned according to the layout shown in Figure 8. The remaining 20 channels utilized temperature sensors uniformly distributed over the spindle housing surface; their positions could be flexibly selected. All sensors were calibrated to ensure measurement consistency, and the uncertainty of temperature measurements was within ±0.3 °C. Experimental data were recorded every 10 s.

### 4.2. Validation of the Effectiveness of the Improved Grey Wolf Algorithm Under Variable Operating Conditions

**Figure 9:**
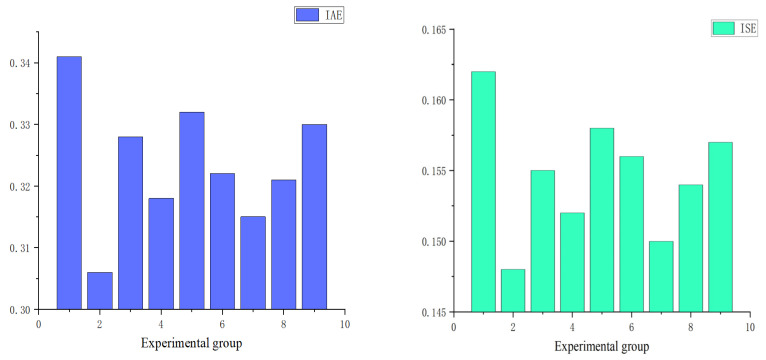
Experimental results of hyperparameter tuning for the IGWO.

First, an orthogonal experimental design was employed to determine the optimal hyperparameter combination for the Improved IGWO. Empirically, the population size ***D*** was set at three levels: 7, 14, and 28. The iteration count ***k*** was also tested at three levels: 50, 100, and 150. The initial value of the control parameter ***a*** was assigned levels of 1.8, 2.0, and 2.2, while its terminal value was tested at 0.01, 0.001, and 0.0001. Subsequently, a standard *L*_9_^($3^{4}$)^ orthogonal array was utilized for combinatorial testing. Randomly selected subsets of 100 data groups from the dataset were used for experimentation, with Integral Square Error (ISE) and Integral Absolute Error (IAE) serving as evaluation metrics. As shown in Figure 9, which presents the experimental results for all hyperparameter combinations, the second experimental group demonstrated the best performance. Consequently, Table 3 lists the optimal hyperparameter combination identified through this orthogonal experiment.

**Figure 10:**
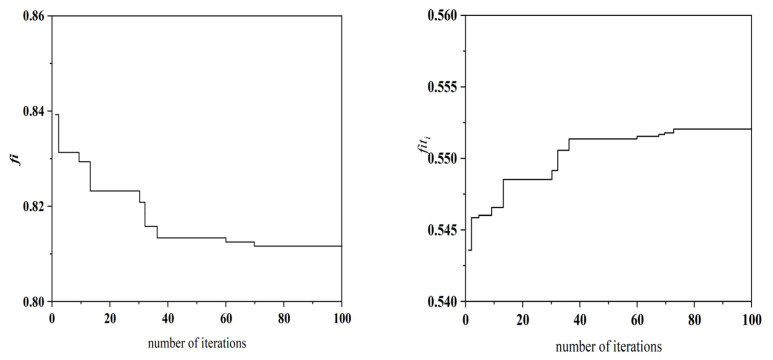
Objective function and fitness change curve.

**Figure 11:**
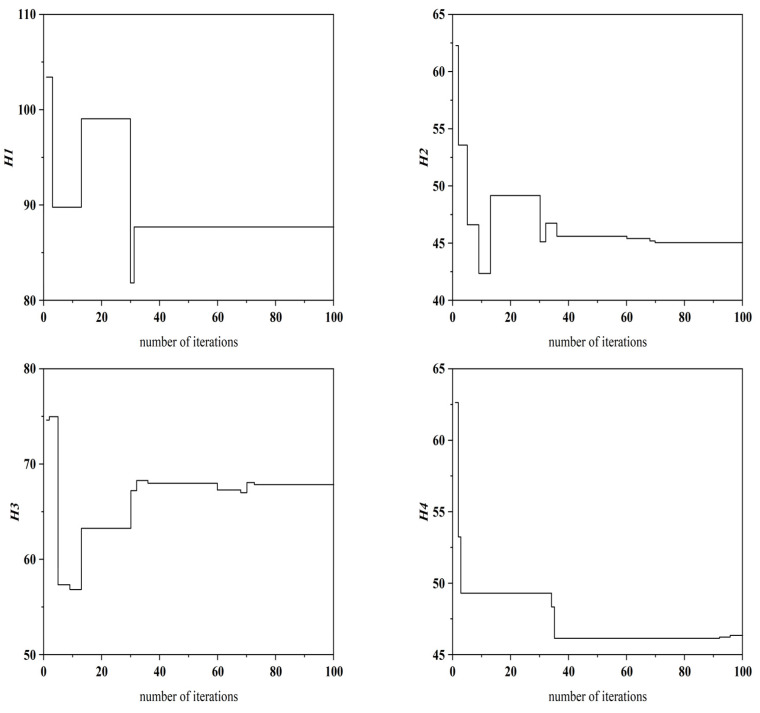

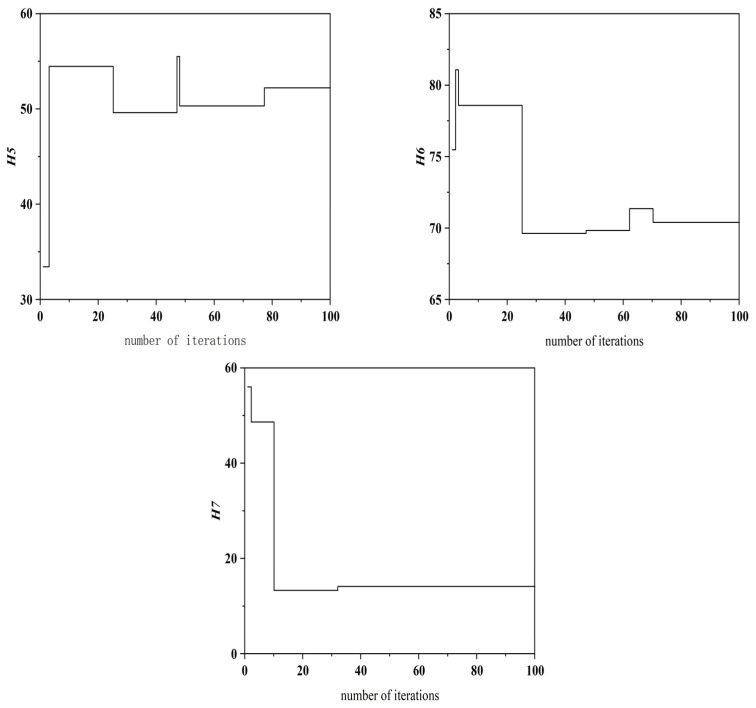
Variation curves of *H*1–*H*7.

(1) Under experimental conditions with an unloaded mechanical spindle rotating at 3000 rpm (*n* = 3000 r) and an environmental temperature of 23.2 °C (*T* = 23.2 °C), seven optimized CHTCs were obtained using the IGWO. The specific values of these parameters and their corresponding locations can be found in Table 4. During the iterative process of the algorithm, the variation trends of the objective function *fᵢ* and the fitness function *fitᵢ* are presented in Figure 10, while the trends for the CHTC (*H*1–*H*7) are shown in Figure 11. Using these seven CHTCs, the resulting objective function value was *fᵢ* = 0.81 and the fitness value was *fitᵢ* = 0.55. As this fitness value represents the highest value achieved during the iterations, the corresponding CHTCs were selected as the final optimized result.

**Figure 12:**
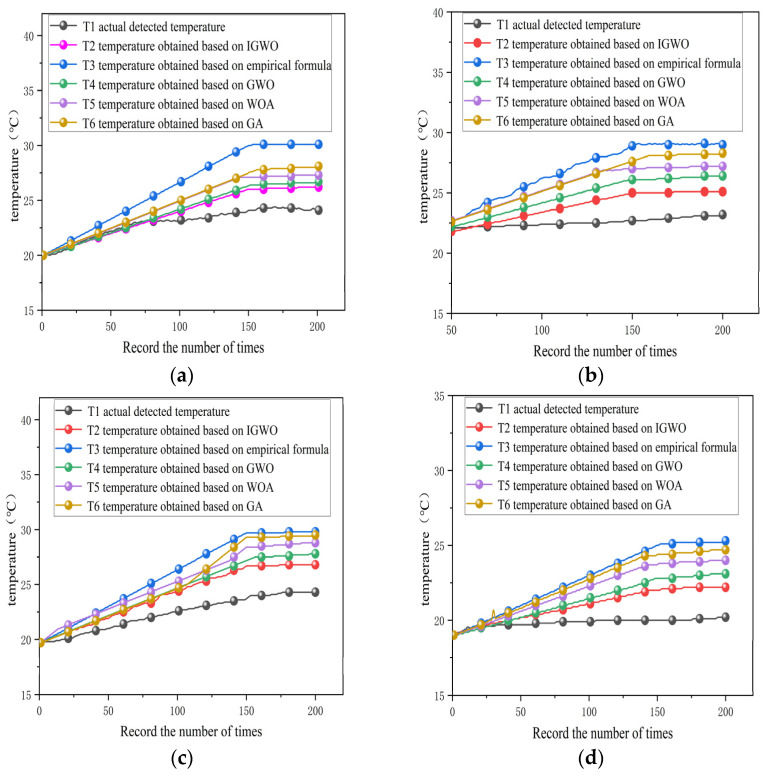
Temperature fitting curve (*n* = 3000 r, *T* = 23.2 °C): (**a**) temperature curve of test point 1, (**b**) temperature curve of test point 3, (**c**) temperature curve of test point 5, (**d**) temperature curve of test point 7.

The performance of the conventional empirical formula method, GWO, WOA, GA, and the proposed IGWO was compared using test points 1, 3, 5, and 7 at the front and rear bearings as examples. Based on the detailed data provided in Table 5, Table 6, Table 7, Table 8 and Table 9, a comparative analysis of different parameter optimization methods in transient temperature field simulations clearly demonstrates the significant advantages of IGWO. As shown in Figure 12.

When the CHTC was optimized using IGWO before conducting transient temperature field analysis, the results showed a high degree of consistency with the actual temperature values. Specifically, the maximum temperature errors at test points 1, 3, 5, and 7 were 2.01 °C, 2.50 °C, 2.22 °C, and 2.10 °C, respectively. The average errors at these four points were 1.11 °C, 1.54 °C, 1.25 °C, and 1.10 °C, all of which remained at a relatively low level. Furthermore, the root Mean Square Errors (MSE) of 0.43 °C, 0.69 °C, 0.58 °C, and 0.53 °C, respectively, indicate a high concentration of data distribution, reflecting the stability and reliability of the temperature field analysis results.

In contrast, significant errors are observed when the empirical formula method, GWO, WOA, and GA are employed for CHTC calculation and temperature field analysis. When the empirical formula method is used, the maximum temperature errors at test points 1, 3, 5, and 7 are 6.10 °C, 6.21 °C, 5.53 °C, and 5.21 °C, respectively. The corresponding mean errors are 3.30 °C, 3.36 °C, 2.68 °C, and 2.50 °C, with root mean square errors of 1.66 °C, 1.72 °C, 1.39 °C, and 1.53 °C. For the GWO method, the maximum temperature errors at the four test points are 2.41 °C, 3.20 °C, 3.53 °C, and 2.95 °C. The mean errors are 1.41 °C, 1.68 °C, 1.46 °C, and 1.53 °C, while the RMSE values are 0.57 °C, 0.79 °C, 0.66 °C, and 0.51 °C. With the WOA method, the maximum errors reach 3.25 °C, 4.05 °C, 4.57 °C, and 3.82 °C. The mean errors are 1.83 °C, 2.07 °C, 1.89 °C, and 1.91 °C, and the root mean square errors values are 0.74 °C, 0.92 °C, 0.71 °C, and 0.69 °C. When using GA, the maximum temperature errors are 3.83 °C, 5.16 °C, 5.24 °C, and 4.57 °C. The mean errors are 2.53 °C, 2.62 °C, 2.26 °C, and 2.15 °C, with root mean square errors values of 1.13 °C, 1.36 °C, 1.25 °C, and 1.32 °C. These results indicate that all four methods exhibit high dispersion and relatively poor stability in the analysis.

(2) Under the experimental conditions of a mechanical spindle load of 30 N/m^2^, operational speed of 4000 rpm (*n* = 4000 r/min), and environmental temperature of 21.8 °C (*T* = 21.8 °C), seven optimized CHTCs were obtained by applying an improved grey wolf optimization algorithm. The specific values and corresponding locations of these parameters are provided in Table 10. Using these seven CHTC values, the objective function reached *f_i_* = 0.817, and the fitness function achieved *fit_i_* = 0.551. This fitness value represents the peak value observed during the iteration process; thus, the current set of CHTC was selected as the final optimization result.

**Figure 13:**
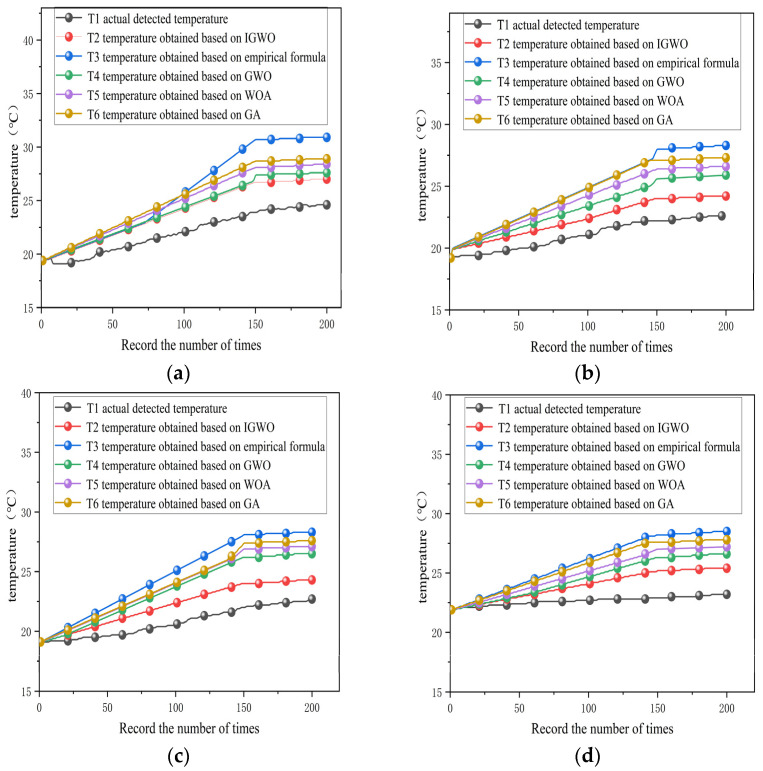
Temperature fitting curve (*n* = 4000 r, *T* = 21.8 °C): (**a**) temperature curve of test point 1, (**b**) temperature curve of test point 3, (**c**) temperature curve of test point 5, (**d**) temperature curve of test point 7.

The performance of the conventional empirical formula method, GWO, WOA, GA, and the proposed IGWO was compared using test points 1, 3, 5, and 7 at the front and rear bearings as examples. Based on the detailed data provided in Table 11, Table 12, Table 13, Table 14 and Table 15, a comparative analysis of different parameter optimization methods in transient temperature field simulations clearly demonstrates the significant advantages of IGWO. As shown in Figure 13.

When the CHTC was optimized using IGWO before conducting transient temperature field analysis, the results showed a high degree of consistency with the actual temperature values. Specifically, the maximum temperature errors at test points 1, 3, 5, and 7 were 2.43 °C, 2.18 °C, 2.39 °C, and 2.22 °C, respectively. The average errors at these four points were 1.21 °C, 1.43 °C, 1.29 °C, and 1.16 °C, all of which remained at a relatively low level. Furthermore, the root Mean Square Errors (MSE) of 0.45 °C, 0.61 °C, 0.59 °C, and 0.49 °C, respectively, indicate a high concentration of data distribution, reflecting the stability and reliability of the temperature field analysis results.

In contrast, significant errors are observed when the empirical formula method, GWO, WOA, and GA are employed for CHTC calculation and temperature field analysis. When the empirical formula method is used, the maximum temperature errors at test points 1, 3, 5, and 7 are 6.33 °C, 5.71 °C, 5.24 °C, and 5.36 °C, respectively. The corresponding mean errors are 3.32 °C, 3.16 °C, 2.57 °C, and 2.53 °C, with root mean square errors of 1.69 °C, 1.70 °C, 1.41 °C, and 1.54 °C. For the GWO method, the maximum temperature errors at the four test points are 3.83 °C, 4.08 °C, 4.63 °C, and 4.07 °C. The mean errors are 1.87 °C, 2.09 °C, 1.91 °C, and 2.01 °C, while the RMSE values are 0.76 °C, 0.94 °C, 0.72 °C, and 0.73 °C. With the WOA method, the maximum errors reach 3.25 °C, 4.05 °C, 4.57 °C, and 3.82 °C. The mean errors are 1.83 °C, 2.07 °C, 1.89 °C, and 1.91 °C, and the root mean square errors values are 0.74 °C, 0.92 °C, 0.71 °C, and 0.69 °C. When using GA, the maximum temperature errors are 4.37 °C, 4.71 °C, 4.92 °C, and 4.68 °C. The mean errors are 2.72 °C, 2.42 °C, 2.04 °C, and 2.17 °C, with root mean square errors values of 1.26 °C, 1.19 °C, 1.21 °C, and 1.30 °C. These results indicate that all four methods exhibit high dispersion and relatively poor stability in the analysis.

The IGWO objectively and accurately optimizes the CHTC by learning and mining large-scale data, thereby effectively avoiding subjective bias. This approach fundamentally enhances the accuracy of transient temperature field analysis and provides a more reliable theoretical basis and data support for related engineering applications.

**Figure 14:**
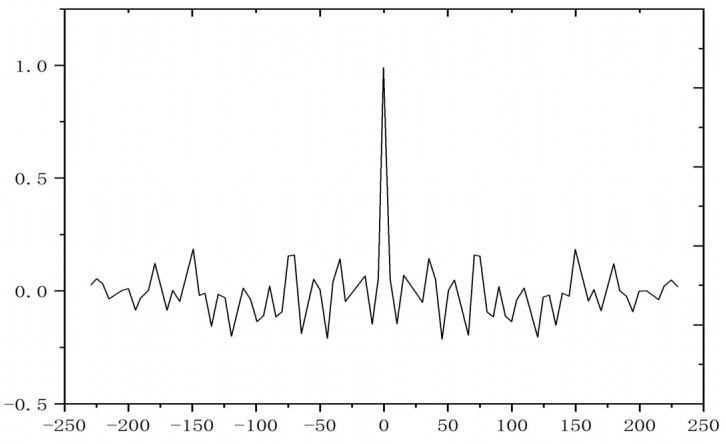
Autocorrelation distribution of temperature deviation.

To evaluate the practical fitting performance of the mechanical spindle temperature model, this study employs autocorrelation function analysis to validate the optimized CHTC model based on IGWO. As shown in Figure 14, the autocorrelation function curve depicts the normalized deviation sequence between the model-calculated temperatures and the measured data. The results indicate that the autocorrelation coefficients of this normalized deviation sequence all fall within the 95%.

The experimental results fully demonstrate the significant advantages of the intelligent soft-sensing method based on the IGWO. These optimized parameters substantially enhance the accuracy and reliability of transient temperature field analysis. Consequently, this method is better suited for engineering scenarios demanding high-precision temperature field prediction. While empirical formula-based methods are simple to operate and computationally inexpensive, they are limited by subjective empirical knowledge and simplified models. Therefore, they are only suitable for preliminary estimation scenarios where lower accuracy is acceptable. Thus, in practical engineering applications, the data-driven IGWO algorithm can effectively meet the requirements for high-precision temperature field analysis, providing a more reliable basis for engineering decision-making.

## 5. Conclusions

This study proposes an intelligent soft-sensor approach based on an IGWO algorithm to address accuracy challenges in measuring the CHTC of CNC machine tool spindles. By integrating an adaptive weight adjustment mechanism and a dynamic disturbance strategy, the optimized algorithm improves the dynamic identification accuracy of the CHTC and exhibits good adaptability under complex dynamic operating conditions.

The main contributions of this work are as follows:

1. **A soft-sensor method based on an intelligent optimization algorithm:** This approach enables accurate dynamic identification of spindle CHTC through the optimization algorithm, overcoming limitations of conventional methods under variable operating conditions.
2. **Innovative adaptive weight adjustment and dynamic disturbance strategies:** These mechanisms enhance the algorithm’s global search ability and convergence speed, allowing the model to respond to spindle variations under complex conditions and further improving the prediction accuracy of the CHTC.
3. **A potential technical pathway for thermal error compensation:** By supporting accurate spindle CHTC estimation, this study offers a transferable framework that may facilitate thermal error compensation in CNC machine tools, showing promise for engineering applications.

Although the proposed method demonstrates robust performance across multiple operating scenarios, several limitations should be noted. First, while the IGWO algorithm performs well under most conditions, its convergence rate may decline, or it may converge to local optima under extreme parameter settings. Second, although the method incorporates data from multiple sensor types, real-time performance and computational efficiency still require further improvement. Moreover, since experimental validation was mainly conducted under simulated conditions, real-world factors such as environmental noise and sensor measurement errors may affect model accuracy. Therefore, additional validation in broader industrial environments is necessary to substantiate its practical applicability.

The main limitation of this study lies in the fact that the validation experiments were conducted solely in a simulated laboratory environment. The robustness of the proposed method against cumulative disturbances in real CNC machining—such as long-term sensor drift, workshop vibrations, and thermal/force disturbances during cutting—has not been evaluated, although these factors directly affect its practical performance in industrial settings. Therefore, future research may consider the following directions:

1. **Collaborative field validation with manufacturing enterprises:** Continuous 24 h production data should be collected from industrial CNC machine tools to assess the method’s performance under actual disturbances.
2. **Development of a hybrid anti-disturbance system:** This system would combine sensor-level calibration (to reduce drift) and algorithm-level denoising techniques, such as integrating wavelet transforms into IGWO, to enhance the method’s resistance to interference.
3. **Cross-process validation:** Testing should be carried out in various machining scenarios, including milling, turning, and grinding, to ensure the method’s generalizability across diverse CNC machining applications.
4. **Further optimization of the algorithm:** To improve real-time performance and convergence speed, adaptive optimization strategies that respond to dynamic changes can be introduced, thereby strengthening the model’s robustness under extreme working conditions.
5. **Data processing and fusion methods:** By refining data preprocessing techniques and algorithmic design, the model’s adaptability to varying working conditions can be improved, along with its ability to identify diverse heat exchange phenomena.

Overall, the proposed intelligent soft-sensor method, based on the IGWO algorithm, successfully addresses CHTC measurement accuracy challenges and provides a novel technical solution for spindle thermal error compensation. While certain challenges remain, this study offers valuable insights for future research in thermal management and error compensation within high-precision manufacturing.

## Figures and Tables

**Table 1:** Single peak and multi-peak benchmark functions.

Test Function	Dimension	Search Scope	Search Scope
$F_{1}(x)=\sum_{i=1}^{n}x_{i}^{2}$	30	[−100,100]	0
$F_{2}(x)=\sum_{i=1}^{n}|x_{i}|+\prod_{i=1}^{n}|x_{i}|$	30	[−10,10]	0
$F_{7}(x)=\sum_{i=1}^{n}ix_{i}^{4}+random\left[0,1\right)$	30	[−1.28,1.28]	0
$F_{9}(x)=\sum_{i=1}^{n}\left[x_{i}^{2}-10\cos(2\pi x_{i})+10\right]$	30	[−5.12,5.12]	0

**Table 2:** Correspondence between the improved grey wolf algorithm and solving optimization problems.

Hunting Behavior of Grey Wolves	CHTC
Wolves	Feasible solution for CHTC
The position of the wolf pack	Location of CHTC (*H*1–*H*7)
The three leading wolves in the wolf pack	The fitness value of CHTC
The process of searching, surrounding, and attacking prey	The process of solving problems
The position of the alpha wolf	The optimal solution to the problem

**Table 3:** Orthogonal experimental design for hyperparameter tuning of the IGWO.

Experiment	D	k	Starting from *a*	Termination of *a*
1	7	50	1.8	0.01
2	7	100	2.0	0.001
3	7	150	2.2	0.0001
4	14	50	2.0	0.0001
5	14	100	2.2	0.01
6	14	150	1.8	0.001
7	28	50	2.2	0.001
8	28	100	1.8	0.0001
9	28	150	2.0	0.01

**Table 4:** CHTC of each part of the main shaft (*n* = 3000 r, *T* = 23.2 °C).

Location of CHTC	CHTC Value
H1	88.21
H2	46.32
H3	68.49
H4	46.72
H5	52.13
H6	70.23
H7	16.54

**Table 5:** Error analysis of predicted temperature using IGWO (*n* = 3000 r, *T* = 23.2 **°**C).

Test Points	ME (°C)	MAE (°C)	MSE (°C)
1	2.01	1.11	0.43
3	2.50	1.54	0.69
5	2.22	1.25	0.58
7	2.10	1.10	0.53

**Table 6:** Error analysis of predicted temperature using GWO (*n* = 3000 r, *T* = 23.2 **°**C).

Test Points	ME (°C)	MAE (°C)	MSE (°C)
1	2.41	1.41	0.57
3	3.20	1.68	0.79
5	3.53	1.46	0.66
7	2.95	1.53	0.51

**Table 7:** Error analysis of predicted temperature using WOA (*n* = 3000 r, *T* = 23.2 °C).

Test Points	ME (°C)	MAE (°C)	MSE (°C)
1	3.25	1.83	0.74
3	4.05	2.07	0.92
5	4.57	1.89	0.71
7	3.28	1.91	0.69

**Table 8:** Error analysis of predicted temperature using GA (*n* = 3000 r, *T* = 23.2 °C).

Test Points	ME (°C)	MAE (°C)	MSE (°C)
1	3.83	2.53	1.13
3	5.16	2.62	1.36
5	5.24	2.26	1.25
7	4.57	2.15	1.32

**Table 9:** Error analysis of temperature derived from empirical formula (*n* = 3000 r, *T* = 23.2 °C).

Test Points	ME (°C)	MAE (°C)	MSE (°C)
1	6.10	3.30	1.66
3	6.21	3.36	1.72
5	5.53	2.68	1.39
7	5.21	2.50	1.53

**Table 10:** CHTC of each part of the main shaft (*n* = 4000 r, *T* = 21.8 °C).

Location of CHTC	CHTC Value
H1	79.75
H2	46.25
H3	61.89
H4	45.31
H5	46.20
H6	68.23
H7	11.12

**Table 11:** Error analysis of predicted temperature using IGWO (*n* = 4000 r, *T* = 21.8 °C).

Test Points	ME (°C)	MAE (°C)	MSE (°C)
1	2.43	1.21	0.45
3	2.18	1.43	0.61
5	2.39	1.29	0.59
7	2.22	1.16	0.49

**Table 12:** Error analysis of predicted temperature using GWO (*n* = 4000 r, *T* = 21.8 °C).

Test Points	ME (°C)	MAE (°C)	MSE (°C)
1	3.12	1.56	0.62
3	3.45	1.72	0.86
5	4.01	1.53	0.73
7	3.49	1.67	0.59

**Table 13:** Error analysis of predicted temperature using WOA (*n* = 4000 r, *T* = 21.8 °C).

Test Points	ME (°C)	MAE (°C)	MSE (°C)
1	3.83	1.87	0.76
3	4.08	2.09	0.94
5	4.63	1.91	0.72
7	4.07	2.01	0.73

**Table 14:** Error analysis of predicted temperature using GA (*n* = 4000 r, *T* = 21.8 °C).

Test Points	ME (°C)	MAE (°C)	MSE (°C)
1	4.37	2.72	1.26
3	4.71	2.42	1.19
5	4.92	2.04	1.21
7	4.68	2.17	1.30

**Table 15:** Error analysis of temperature derived from empirical formula (*n* = 4000 r, *T* = 21.8 °C).

Test Points	ME (°C)	MAE (°C)	MSE (°C)
1	6.33	3.32	1.69
3	5.71	3.16	1.70
5	5.24	2.57	1.41
7	5.36	2.53	1.54

## Data Availability

The original contributions presented in the study are included in the article, further inquiries can be directed to the corresponding author.

## Abbreviations

The following variables are used in this manuscript:

Variable	Variable Name
H1	Heat transfer coefficient between the rear axle pulley and the outside air
H2	Rear bearing right end face and air gap heat transfer coefficient
H3	Heat transfer coefficient between the intermediate bearing and the air gap
H4	Heat transfer coefficient between the left end face of the rear bearing and the air gap
H5	Heat transfer coefficient between the front axle shoulder and the outside air
H6	Heat transfer coefficient between the shell and the air gap
H7	Heat transfer coefficient between the shell and the outside air
*H*	Heat Transfer Coefficient
*λ*	Thermal Conductivity of Air
*N_u_*	Nusselt Number
*l*	Characteristic Length
*ΔT*	The temperature difference between the spindle surface and the air
Re	Reynolds Number
*n*	speed
*r*	Bearing Radius
*d*	Bearing Diameter
*v*	linear velocity
*μ*	Kinematic Viscosity of Air
Pr	Prandtl Number
*T_i_*	environment temperature
*T*_0_	Transient temperature field of the spindle
*α**,**β**,**δ**,**ω*	The level of wolf packs
*A*	Coefficient Vector A
*C*	Coefficient Vector C
*D*_0_	The distance measurement between the individual gray wolf and its prey is based on the coefficient C.
*r_1_*	random vector r_1_
*r_2_*	random vector r_2_
*ω_i_*	weight factors
*Xp(t)*	Indicate the location of the prey
*X(t)*	The position of the gray wolf individual at the t-th generation
*P*	Disturbance probability
*G*	The dimension of the optimization problem
*k*	number of iterations
*a*	Control parameters
*M*	An individual, after a disturbance
*lb*	Lower bound of the individual gray wolf’s position
*ub*	Upper bound of an individual gray wolf’s position
*r_3_*	random vector r_3_
*Ω*	spatial function
*T_s_*	Actual measured temperature
*T_y_*	Temperature obtained from finite element simulation
*f_i_*	objective function
*fit_i_*	Fitness function
*Tx(t)*	Instantaneous values of each node on the main axis
*E*	Heat Capacity Matrix
*K*	Thermal Conductivity Matrix
*Z*	Temperature load matrix
$T^{\prime }$	Node temperature array
*∆t*	Time step
$\dot{T}$	Derivative array of node temperature with respect to time
